# EPIGENETIC MECHANISMS OF TISSUE AGING

**DOI:** 10.1093/geroni/igac059.106

**Published:** 2022-12-20

**Authors:** Payel Sen, Na Yang, James Occean, Daniel Melters, Changyou Shi, Lin Wang, Simone Sidoli, Yamini Dalal

**Affiliations:** National Institute on Aging, NIH, Baltimore, Maryland, United States; NIA, Baltimore, Maryland, United States; NIA, Baltimore, Maryland, United States; NCI, Bethesda, Maryland, United States; NIA, Baltimore, Maryland, United States; NIH, Bethesda, Maryland, United States; Albert Einstein College of Medicine, New York, New York, United States; NCI, Bethesda, Maryland, United States

## Abstract

Epigenetic alterations are one of the key hallmarks of aging. How epigenetic changes broadly affect euchromatin-heterochromatin balance, three-dimensional chromatin conformation, histone composition and global transcriptome in aging have not been addressed. Using murine liver as a model organ and an integrated multi-omics approach, we find that aged cells assume a repressed chromatin state characterized by condensed chromatin, broad heterochromatinization, global suppression of the transcriptome, and inability to self-interact over long distances. Our findings reveal that global chromatin alterations pose a barrier and promote a cellular state that is refractory to injury sensing and repair.

